# Dynamic anti-plane behavior of rare earth giant magnetostrictive medium with a circular cavity defect in semi-space

**DOI:** 10.1038/s41598-021-92841-5

**Published:** 2021-06-29

**Authors:** Zhiwei Liu, Hui Qi

**Affiliations:** 1 Nuclear Power Institute of China, Chengdu, China; 2 College of Aerospace and Civil Engineering, Harbin Engineering University, Harbin, China

**Keywords:** Mechanical engineering, Materials science, Materials for devices, Structural materials, Theory and computation

## Abstract

An analytical solution to the anti-plane dynamics problem of semi-space rare earth giant magnetostrictive media with circular cavity defects near the horizontal boundary under the action of SH wave is studied. Based on the Helmholtz theorem and the theory of complex function, the elastic-magnetic dynamic equation of magnetostrictive medium is established, and the semi-space incident wave field is written. In addition, based on the theory of complex function and the method of wave function expansion, the expression of the wave function of the scattered displacement field and the corresponding magnetic potential of the scattered wave under the condition of no stress and magnetic insulation of the horizontal boundary are obtained. Then, based on the conditions of free boundary stress, continuous magnetic induction intensity and continuous magnetic potential around the circular cavity, the infinite linear algebraic equations are established. Finally, the analytical expressions of dynamic stress concentration factor and magnetic field intensity concentration factor around circular cavity in semi-space rare earth giant magnetostrictive medium are obtained. Numerical examples show that the analysis results depend on the following parameters: permeability, dimensional-piezomagnetic coefficient, frequency of the incident wave, incident angle, distance between the circular cavity and horizontal boundary. These results have certain reference value for the study of non-destructive testing and failure analysis of rare earth giant magnetostrictive materials.

## Introduction

Rare earth giant magnetostrictive materials, as a new type of intelligent material with strategic characteristics in the twenty-first century, are widely regarded in the world. Rare earth giant magnetostrictive material has a big magnetostrictive strain ratio, which has been proved to be superior to piezoelectric material. So it is a high-tech product for both military and civil use. It is expected that the main applications of the materials in the future will be as follows: defence industry and aerospace, marine science and offshore engineering, machinery and automotive manufacturing, high-power ultrasound and medical services. China has basically reached the international advanced level in the manufacture of the rare earth giant magnetostrictive materials, but the theoretical research of mechanical properties is still in a backward stage. As a large country of rare earth resources, China's resource advantages have not been fully brought into play and applied. Due to the problem of magnetoelastic coupling, natural factors, or the formation of many different defects in the processing and manufacturing process, such as irregular circular cavity in shape, inclusions of different medium parameters, small cracks and defects separated from the medium are widely found in various giant magnetostrictive elements. When the magnetostrictive material enters the working state, it will produce mechanical vibration inside the material, and the scattering phenomenon will occur when the mechanical wave encounters defects or interfaces in the process of internal propagation of the material, which will cause displacement mutation or stress concentration. It will have an unnegligible influence on the strength of giant magnetostrictive materials. Engineering practice has also proved that the destruction of giant magnetostrictive materials began from these tiny defects and interfaces. In recent years, great attention has been paid to the problems of internal defects, boundary and interface damage of magnetostrictive materials at home and abroad, but the research results are relatively few. Therefore, the research of this paper is extremely urgent. When the circular cavity defect exists near the boundary, the anti-plane dynamic problems will be more complex than that of the elastic materials.

In recent years, many valuable achievements have been made in the study of SH wave propagation in elastic and piezoelectric media. Qi^1^ studied the scattering problem of SH wave propagation in two-material semi-space with cylindrical inclusions near the interface by using Green's function and complex function method. Zhao^2^ studied the scattering problem of SH wave propagation in the middle of a two-phase space with circular hole and cylindrical inclusions on the horizontal interface. Zhang^3,4^ studied the scattering of SH waves by cylindrical inclusions and semi-cylindrical holes in the semi-space of bi-phase media by using Green's function method and complex function method, and obtained the steady-state solution of the problem and then analyzed the anti-plane dynamics of waves caused by interface cracks in bi-phase piezoelectric media. Shindo^5^ studied the dynamics of an elliptical cylindrical piezoelectric inclusion in an infinite piezoelectric medium by using the wave function expansion method. Feng^6^ studied partial elliptic cylindrical piezoelectric inclusions in piezoelectric medium by using wave function expansion method and singular integral equation technique. Sahu^7^ used the elastic wave theory to study the transference of Love-type waves in functionally graded piezoelectric material layer bonded between viscous liquid and pre-stressed piezoelectric half-space. Chaudhary^8^ adopted the analytical approach to investigate the SH waves in a composite structure consisting of initially stressed rotating piezoelectric layer and initially stressed substrate with rotation.

Recently, scholars at home and abroad have carried out some numerical, experimental and simulation analysis on giant magnetostrictive materials to study the propagation of coupled magnetoelastic waves. In the piezomagnetic fracture mechanics problem, the problem of magnetoelastic coupling in the rare earth giant magnetostrictive media needs to be paid more attention. The rare earth giant magnetostrictive materials exhibit significant magnetostrictive effects. So, it is necessary to consider the effect of magnetostrictive effect on the deformation of such materials. Chaudhary^9,10^ computed the normal and shear stresses, dielectric, and electric pontential in an irregular initially stressed piezoelectric substrate with a irregularity depression under the moving load. Then proposed an analytical technique to study the shear horizontal wave propagation in a layered pre-stressed rotating cylindrical tube structure consisting of a piezoelectric material layer which is wrapped on piezomagnetic material having an imperfect interface. Singhal^11,12^ studied the propagation of Love-type wave in functionally graded piezoelectric material layer bonded between piezomagnetic plate and pre-stressed piezoelectric half-space and then used liouville-green approximation to study the surface waves in functionally graded piezoelectric material clubbed between two dissimilar piezomagnetic media. Jiang^13^ used the decoupling technique and wave function method to study the dynamic behavior around the circular hole in exponential gradient piezomagnetic material. Pang^14^ used liner magneto-electro-elastic spring model to study the scattering problem of SH wave propagation in the layered piezoelectric and piezomagnetic plates with nonideal magneto-elastic interface. Ray^15^ established the magneto-elastic dynamic equation, and by using the Green’s function method, the characteristics of the wave propagation behavior of the piezomagnetic material layer on the functional gradient piezomagnetic material under the action of the interface pulse point source had been revealed. Guo^16^ studied the scattering problem of SH wave propagation in layered piezoelectric/piezomagnetic medium with cylinders when SH wave meets the functionally graded interlayers. Kong^17^ studied the scattering behavior of SH wave in the coupled structures of piezomagnetic substrates and orthogonal piezoelectric layers under different shear directions. Ezzin^18^ used the ordinary differential equations and stiffness matrix method to study the scattering problem of SH wave propagation in piezomagnetic/piezoelectric laminates. Wei^19^ presented a numerical solution to the problem of SH wave scattering in the piezoelectric/piezomagnetic plate with a non-ideal interface. Liu^20^ studied the propagation of SH wave in periodic layered piezomagnetic structure, then concluded that the band gap characteristics of magnetic closure and opening were the same. Ebrahimi^21^ used the nonlocal elasticity theory to study the bending of magneto-electric-elastic nanobeams.

In the previous research work, the influence of boundary or defect on the propagation of elastic wave in magnetostrictive materials has been discussed respectively in relevant studies. However, when the internal defect and external boundary of the material exist simultaneously and interact, the anti-plane dynamics problem of magnetostrictive material needs further theoretical analysis. So, in this article, the physical model is simplified into a semi-space rare earth giant magnetostrictive medium with a circular cavity. We established the elastic-magnetic dynamic equation of the rare earth giant giant magnetostrictive medium. Then the scattered wave field inside the material can be constructed by using the complex function and Green's function method. According to the boundary conditions of circular cavity, the dynamic stress concentration factor and magnetic field intensity concentration factor of the rare earth giant magnetostrictive material are obtained by numerical examples. The purpose of this paper is to provide an effective theoretical analysis method for the damage and failure of rare earth giant magnetostrictive materials in engineering practice.

## Theoretical analysis

### Problem description

**Figure 1:**
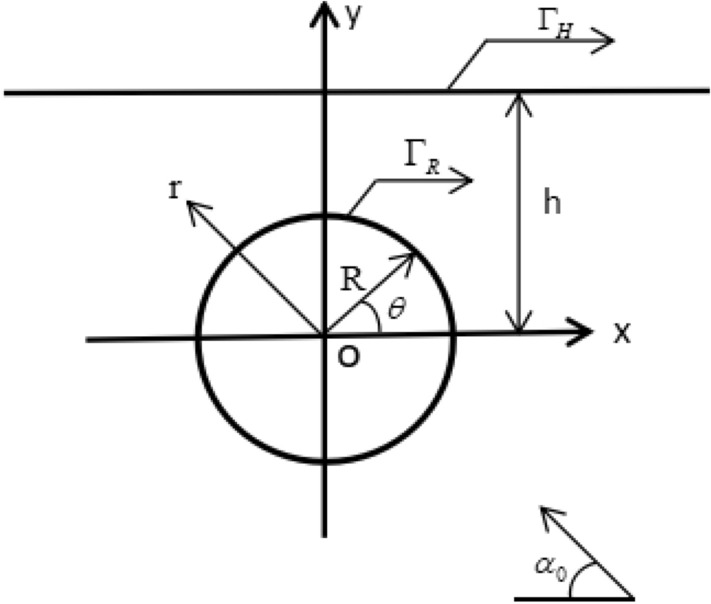
Semi-space model with a circular cavity.

Figure 1 shows the physical model. In the semi-space rare earth giant magnetostrictive medium, there is a circular cavity with a radius of $$R$$, and the shallow depth is $$h$$$$\left( {h \ge R} \right)$$. The elastic constant, mass density, permeability and piezomagnetic coefficient of rare earth giant magnetostrictive medium are $$c_{{44}}$$, $$\rho$$, $$\mu _{{{\text{11}}}}$$ and $$h_{{15}}$$, respectively. The horizontal boundary is $$\Gamma _{H}$$. The distance between the horizontal boundary $$\Gamma _{H}$$ and the central position of the circular cavity is $$h$$. The boundary of the circular cavity is $$\Gamma _{R}$$. The center point of the rectangular coordinate system $$\left( {o,x,y} \right)$$ coincides with the center of the circular cavity. A group of steady-state incident plane SH wave $$w^{i}$$ (incident wave function) is incident along the direction of $$\alpha _{{\text{0}}}$$ angle negative to the X-axis. It produces a series of reflected waves $$w^{r}$$ (reflected wave function) with the semi-space boundary, and the interaction with the circular cavity will produce scattered waves $$w^{s}$$ (scattered wave function).

Then, according to the physical model diagram, we can determine the boundary conditions of the semi-space rare earth giant magnetostrictive medium, which can be expressed as:

1 $$ \begin{aligned}    & \Gamma _{H} :\tau _{{yz}}  = 0,B_{y}  = 0(y = h) \\     & \Gamma _{R} :\tau _{{rz}}  = 0,B_{r}  = B_{r}^{c} ,\phi  = \phi ^{c} (r = R), \\  \end{aligned} $$

where, $B_{r}$ and $B_{r}^{c}$ respectively represent the magnetic induction intensity in the rare earth giant magnetostrictive medium and magnetic induction intensity in the circular cavity. $\phi$ and $\phi ^{c}$ represent the magnetic potential in the rare earth giant magnetostrictive medium and magnetic potential in the circular cavity. The superscript "c" represents the physical quantity in the circular cavity.

### Governing equations and constitutive equations

In this paper, let the Z-axis be the magnetic polarization direction of the rare earth giant magnetostrictive material, then the constitutive equations describing its linear magnetoelastic coupling are Eq. (2) and the steady-state governing equations (ignoring the time factor) of the anti-plane dynamics problem are Eq. (3).

2 $$ \begin{aligned}    & \sigma _{\beta }  = c_{{44}} \frac{{\partial w}}{{\partial x_{\beta } }} + h_{{15}} \frac{{\partial \phi }}{{\partial x_{\beta } }} \\     & B_{\beta }  = h_{{15}} \frac{{\partial w}}{{\partial x_{\beta } }} - \mu _{{11}} \frac{{\partial \phi }}{{\partial x_{\beta } }}\quad \left( {\beta  = 1,2} \right), \\  \end{aligned} $$

3 $$ \begin{aligned}    & c_{{44}} \nabla ^{2} w + h_{{15}} \nabla ^{2} \phi  + \rho \omega ^{2} w = 0 \\     & h_{{15}} \nabla ^{2} w - \mu _{{11}} \nabla ^{2} \phi  = 0, \\  \end{aligned} $$

where, $c_{{44}}$, $\mu _{{{\text{11}}}}$ and $h_{{15}}$ are respectively the elastic constant, permeability and piezomagnetic coefficient. $w$, $\omega$, $\rho$ and $\phi$ respectively represent the plane displacement, circular frequency of SH wave, mass density and magnetic potential in the medium. $\nabla ^{{\text{2}}}$ is the Laplace operator. Let $\phi  = {{h_{{15}} \left( {\omega  + f} \right)} \mathord{\left/ {\vphantom {{h_{{15}} \left( {\omega  + f} \right)} {\mu _{{11}} }}} \right. \kern-\nulldelimiterspace} {\mu _{{11}} }}$, then Eq. (3) can be simplified as follows:

4 $$ \begin{aligned}    & \nabla ^{{\text{2}}} w + k^{2} w = 0 \\     & \nabla ^{2} f = 0, \\  \end{aligned} $$

where, $k$ is the wave number and $k^{2}  = \rho \omega ^{{\text{2}}} {\text{/}}c^{*} ,c^{*}  = c_{{44}}  + h^{2} _{{15}} /\mu _{{11}}$.

Its constitutive equations are expressed as:

5 $$ \begin{aligned}    & \tau _{{xz}}  = c_{{44}} \frac{{\partial w}}{{\partial x}} + h_{{15}} \frac{{\partial \varphi }}{{\partial x}} \\     &  \tau _{{yz}}  = c_{{44}} \frac{{\partial w}}{{\partial y}} + h_{{15}} \frac{{\partial \varphi }}{{\partial y}} \\     &  B_{x}  = h_{{15}} \frac{{\partial w}}{{\partial x}} - \mu _{{11}} \frac{{\partial \varphi }}{{\partial x}} \\     & B_{y}  = h_{{15}} \frac{{\partial w}}{{\partial y}} - \mu _{{11}} \frac{{\partial \varphi }}{{\partial y}}. \\  \end{aligned} $$

By introducing polar coordinates, the governing Eq. (4) can be expressed as:

6 $$ \begin{aligned}    & \frac{{\partial ^{{\text{2}}} w}}{{\partial r^{2} }} + \frac{1}{r}\frac{{\partial w}}{{\partial r}} + \frac{1}{{r^{2} }}\frac{{\partial ^{2} w}}{{\partial \theta ^{2} }} + k^{2} w = 0 \\     & \frac{{\partial ^{2} f}}{{\partial r^{2} }} + \frac{1}{r}\frac{{\partial f}}{{\partial r}} + \frac{1}{{r^{2} }}\frac{{\partial ^{2} f}}{{\partial \theta ^{2} }} = 0. \\  \end{aligned} $$

The constitutive Eq. (5) can be expressed as:

7 $$ \begin{aligned}    & \tau _{{rz}}  = c_{{44}} \frac{{\partial w}}{{\partial r}} + h_{{15}} \frac{{\partial \varphi }}{{\partial r}} \\     & \tau _{{\theta z}}  = c_{{44}} \frac{1}{r}\frac{{\partial w}}{{\partial \theta }} + h_{{15}} \frac{1}{r}\frac{{\partial \varphi }}{{\partial \theta }} \\     & B_{r}  = h_{{15}} \frac{{\partial w}}{{\partial r}} - \mu _{{11}} \frac{{\partial \varphi }}{{\partial r}} \\     & B_{\theta }  = h_{{15}} \frac{1}{r}\frac{{\partial w}}{{\partial \theta }} - \mu _{{11}} \frac{1}{r}\frac{{\partial \varphi }}{{\partial \theta }}. \\  \end{aligned} $$

By using the complex function method, the complex plane $\left( {z,\bar{z}} \right)$ is introduced. Then let $z = x + {\text{i}}y,\bar{z} = x - {\text{i}}y$, then the governing Eq. (4) can be expressed in the complex plane $\left( {z,\bar{z}} \right)$ as follows:

8 $$ \begin{aligned}    & \frac{{\partial w}}{{\partial z\partial \bar{z}}} + \frac{1}{4}k^{2} w = 0 \\     & \frac{{\partial ^{2} f}}{{\partial z\partial \bar{z}}} = 0, \\  \end{aligned} $$

Then, in the complex plane, the constitutive equations shown in Eq. (5) can be transformed into:

9 $$ \begin{aligned}    & \tau _{{xz}}  = c_{{44}} \left( {\frac{{\partial w}}{{\partial z}} + \frac{{\partial w}}{{\partial \bar{z}}}} \right) + h_{{15}} \left( {\frac{{\partial \phi }}{{\partial z}} + \frac{{\partial \phi }}{{\partial \bar{z}}}} \right) \\     & \tau _{{yz}}  = ic_{{44}} \left( {\frac{{\partial w}}{{\partial z}} - \frac{{\partial w}}{{\partial \bar{z}}}} \right) + ih_{{15}} \left( {\frac{{\partial \phi }}{{\partial z}} - \frac{{\partial \phi }}{{\partial \bar{z}}}} \right) \\     & B_{x}  = h_{{15}} \left( {\frac{{\partial w}}{{\partial z}} + \frac{{\partial w}}{{\partial \bar{z}}}} \right) - \mu _{{11}} \left( {\frac{{\partial \phi }}{{\partial z}} + \frac{{\partial \phi }}{{\partial \bar{z}}}} \right) \\     & B_{y}  = ih_{{15}} \left( {\frac{{\partial w}}{{\partial z}} - \frac{{\partial w}}{{\partial \bar{z}}}} \right) - i\mu _{{11}} \left( {\frac{{\partial \phi }}{{\partial z}} - \frac{{\partial \phi }}{{\partial \bar{z}}}} \right). \\  \end{aligned} $$

By introducing the polar coordinate transformation relation, let $z = re^{{{\text{i}}\theta }} ,\bar{z} = re^{{ - {\text{i}}\theta }}$, the constitutive equations in the complex plane $\left( {z,\bar{z}} \right)$ can be expressed as:

10 $$ \begin{aligned}    & \tau _{{rz}}  = c_{{44}} \left( {\frac{{\partial w}}{{\partial z}}e^{{i\theta }}  + \frac{{\partial w}}{{\partial \bar{z}}}e^{{ - i\theta }} } \right) + h_{{15}} \left( {\frac{{\partial \varphi }}{{\partial z}}e^{{i\theta }}  + \frac{{\partial \phi }}{{\partial \bar{z}}}e^{{ - i\theta }} } \right) \\     & \tau _{{\theta z}}  = ic_{{44}} \left( {\frac{{\partial w}}{{\partial z}}e^{{i\theta }}  - \frac{{\partial w}}{{\partial \bar{z}}}e^{{ - i\theta }} } \right) + ih_{{15}} \left( {\frac{{\partial \phi }}{{\partial z}}e^{{i\theta }}  - \frac{{\partial \phi }}{{\partial \bar{z}}}e^{{ - i\theta }} } \right) \\     & B_{r}  = h_{{15}} \left( {\frac{{\partial w}}{{\partial z}}e^{{i\theta }}  + \frac{{\partial w}}{{\partial \bar{z}}}e^{{ - i\theta }} } \right) - \mu _{{11}} \left( {\frac{{\partial \phi }}{{\partial z}}e^{{i\theta }}  + \frac{{\partial \phi }}{{\partial \bar{z}}}e^{{ - i\theta }} } \right) \\     & B_{\theta }  = ih_{{15}} \left( {\frac{{\partial w}}{{\partial z}}e^{{i\theta }}  - \frac{{\partial w}}{{\partial \bar{z}}}e^{{ - i\theta }} } \right) - i\mu _{{11}} \left( {\frac{{\partial \phi }}{{\partial z}}e^{{i\theta }}  - \frac{{\partial \phi }}{{\partial \bar{z}}}e^{{ - i\theta }} } \right), \\  \end{aligned} $$

where,$\tau _{{rz}}$ and $\tau _{{\theta z}}$ are the radial and tangential stresses of the magnetostrictive medium, and $B_{r}$ and $B_{\theta }$ are the radial and tangential magnetic induction intensities of the circular cavity.

### Incident wave and reflected wave

In the isotropic and uniform semi-space rare earth giant magnetostrictive medium, after removing the time factor, the incident plane displacement field $w^{i}$ and the corresponding magnetic potential of the incident wave $\phi ^{i}$ can be expressed as:

11 $$ \begin{aligned}    & w^{i}  = w_{0} \exp \left\{ {\frac{{ik}}{2}\left[ {\left( {z - ih} \right)e^{{ - i\alpha _{0} }}  + \left( {\bar{z} + ih} \right)e^{{i\alpha _{0} }} } \right]} \right\} \\     & \phi ^{i}  = \frac{{h_{{15}} }}{{\mu _{{11}} }}w^{i} . \\  \end{aligned} $$

Then, in order to satisfy the stress free boundary condition on the horizontal boundary, the constructed reflected displacement field can be expressed as:

12 $$ w^{r}  = w_{0} \exp \left\{ {\frac{{ik}}{2}\left[ {\left( {z - ih} \right)e^{{i\alpha _{0} }}  + \left( {\bar{z} + ih} \right)e^{{ - i\alpha _{0} }} } \right]} \right\}. $$

In order to discuss the magnetic potential corresponding to the reflected wave, according to the governing Eq. (8), the magnetic potential function corresponding to the reflected wave can be expressed as:

13 $$ \phi ^{r}  = \frac{{h_{{15}} }}{{\mu _{{11}} }}\left( {w^{r}  + f^{r} } \right). $$

According to the Laplace equation, we obtain the magnetic potential additional function in the corresponding magnetic potential of the reflected wave as follows:

14 $$ f^{r}  = \sum\limits_{{n =  - \infty }}^{\infty } {A_{n} \left( {z - ih} \right)^{{ - n}}  + B_{n} \left( {\bar{z} + ih} \right)^{{ - n}} } . $$

Since the magnetic potential at infinity can not be infinite, the Eq. (14) can be expressed as:

15 $$ f^{r}  = \sum\limits_{{n = 0}}^{\infty } {A_{n} \left( {z - ih} \right)^{{ - n}}  + B_{n} \left( {\bar{z} + ih} \right)^{{ - n}} } . $$

In order to obtain the unknown coefficients, we take the semi-space rare earth giant magnetostrictive medium without defects as an example to obtain the unknown coefficients in the magnetic potential additional function. The conditions of stress freedom and magnetic insulation should be satisfied at the interface of semi-space, then there are the following:

16 $$ \begin{aligned}    & \tau _{{yz}}  = 0 \\     & B_{y}  = 0. \\  \end{aligned} $$

Let's first discuss the case of stress freedom. In the complex plane $\left( {z,\bar{z}} \right)$, the stress of the incident wave $\tau _{{yz}}^{i}$ is expressed as the Eq. (17) and the stress of the reflected wave is expressed as (18).

17 $$ \tau _{{yz}}^{i}  = i\left( {c_{{44}}  + \frac{{h_{{15}}^{2} }}{{\mu _{{11}} }}} \right)w_{0} k\sin \alpha _{0} \exp \left( {\frac{{ik}}{2}\left[ {\left( {z - ih} \right)e^{{ - i\alpha _{0} }}  + \left( {\bar{z} + ih} \right)e^{{i\alpha _{0} }} } \right]} \right), $$

18 $$ \begin{aligned}   \tau _{{yz}}^{r}  &  =  - i\left( {c_{{44}}  + \frac{{h_{{15}}^{2} }}{{\mu _{{11}} }}} \right)w_{0} k\sin \alpha _{0} \exp \left( {\frac{{ik}}{2}\left[ {\left( {z - ih} \right)e^{{ - i\alpha _{0} }}  + \left( {\bar{z} + ih} \right)e^{{i\alpha _{0} }} } \right]} \right) \\     & \quad  - i\frac{{h_{{15}}^{2} }}{{\mu _{{11}} }}\sum\limits_{{n = 1}}^{{ + \infty }} {n\left[ {A_{n} \left( {z - ih} \right)^{{ - n - 1}}  - B_{n} \left( {\bar{z} + ih} \right)^{{ - n - 1}} } \right]} . \\  \end{aligned} $$

Then the total stress of the semi-space rare earth giant magnetostrictive medium can be expressed as:

19 $$ \begin{aligned}   \tau _{{yz}}  &  = \tau _{{yz}}^{i}  + \tau _{{yz}}^{r}  \\     &  = i\left( {c_{{44}}  + \frac{{h_{{15}}^{2} }}{{\mu _{{11}} }}} \right)w_{0} k\sin \alpha _{0} \exp \left( {\frac{{ik}}{2}\left[ {\left( {z - ih} \right)e^{{ - i\alpha _{0} }}  + \left( {\bar{z} + ih} \right)e^{{i\alpha _{0} }} } \right]} \right) \\     & \quad  - i\left( {c_{{44}}  + \frac{{h_{{15}}^{2} }}{{\mu _{{11}} }}} \right)w_{0} k\sin \alpha _{0} \exp \left( {\frac{{ik}}{2}\left[ {\left( {z - ih} \right)e^{{i\alpha _{0} }}  + \left( {\bar{z} + ih} \right)e^{{ - i\alpha _{0} }} } \right]} \right) \\     & \quad  - i\frac{{h_{{15}}^{2} }}{{\mu _{{11}} }}\sum\limits_{{n = 1}}^{{ + \infty }} {n\left[ {A_{n} \left( {z - ih} \right)^{{ - n - 1}}  - B_{n} \left( {\bar{z} + ih} \right)^{{ - n - 1}} } \right]} . \\  \end{aligned} $$

Substitute the coordinates $z = x + ih,\bar{z} = x - ih$ at the horizontal boundary into $\tau _{{yz}}$, and then get the following formula:

20 $$ \begin{aligned}   \tau _{{yz}}  &  = \tau _{{yz}}^{i}  + \tau _{{yz}}^{r}  \\     &  = i\left( {c_{{44}}  + \frac{{h_{{15}}^{2} }}{{\mu _{{11}} }}} \right)w_{0} k\sin \alpha _{0} \exp \left( {\frac{{ik}}{2}\left[ {xe^{{ - i\alpha _{0} }}  + xe^{{i\alpha _{0} }} } \right]} \right) \\     & \quad  - i\left( {c_{{44}}  + \frac{{h_{{15}}^{2} }}{{\mu _{{11}} }}} \right)w_{0} k\sin \alpha _{0} \exp \left( {\frac{{ik}}{2}\left[ {xe^{{i\alpha _{0} }}  + xe^{{ - i\alpha _{0} }} } \right]} \right) \\     & \quad  - i\frac{{h_{{15}}^{2} }}{{\mu _{{11}} }}\sum\limits_{{n = 1}}^{{ + \infty }} {n\left[ {A_{n} x^{{ - n - 1}}  - B_{n} x^{{ - n - 1}} } \right]}  \\     &  =  - i\frac{{h_{{15}}^{2} }}{{\mu _{{11}} }}\sum\limits_{{n = 1}}^{{ + \infty }} {n\left[ {A_{n} x^{{ - n - 1}}  - B_{n} x^{{ - n - 1}} } \right]} . \\  \end{aligned} $$

Then we discuss the case of magnetic insulation, namely $B_{y}  = 0$, and $B_{y}$ can be expressed as the following formula:

21 $$ B_{y}  = ih_{{15}} \sum\limits_{{n = 1}}^{{ + \infty }} {n\left[ {A_{n} \left( {z - ih} \right)^{{ - n - 1}}  - B_{n} \left( {\bar{z} + ih} \right)^{{ - n - 1}} } \right]} . $$

Substitute the coordinates $z = x + ih,\bar{z} = x - ih$ at the horizontal boundary into $B_{y}$, and then get the following formula:

22 $$ B_{y}  = ih_{{15}} \sum\limits_{{n = 1}}^{{ + \infty }} {n\left[ {A_{n} x^{{ - n - 1}}  - B_{n} x^{{ - n - 1}} } \right]} . $$

It is not difficult to find that the solutions of the two equations are equivalent by combining $\tau _{{yz}}  = 0,B_{y}  = 0$, namely:

23 $$ A_{n} x^{{ - n - 1}}  - B_{n} x^{{ - n - 1}}  = 0. $$

Thus, we find that Eq. (23) is true for any x, so $A_{n}  = B_{n}$ is obtained. Then the magnetic potential additional function corresponding to the reflected wave can be expressed as:

24 $$ f^{r}  = \sum\limits_{{n = 0}}^{{ + \infty }} {A_{n} \left( {z - ih} \right)^{{ - n}} } . $$

Therefore, we represent the total magnetic potential in the semi-space rare earth giant magnetostrictive medium, and the total magnetic potential is expressed as:

25 $$ \begin{aligned}   \phi  &  = \phi ^{i}  + \phi ^{r}  \\     &  = \frac{{h_{{15}} }}{{\mu _{{11}} }}\left( {w^{i}  + w^{r} } \right) + \sum\limits_{{n = 0}}^{{ + \infty }} {A_{n} \left( {z - ih} \right)^{{ - n}} } . \\  \end{aligned} $$

Since when $n = 0,r \to \infty$, $\phi  \to \phi ^{i}$, so we can get $A_{{\text{0}}}  = {\text{0}}$, the formula (24) can be expressed as:

26 $$ \begin{aligned}   \phi  &  = \phi ^{i}  + \phi ^{r}  \\     &  = \frac{{h_{{15}} }}{{\mu _{{11}} }}\left( {w^{i}  + w^{r} } \right) + \sum\limits_{{n = 1}}^{{ + \infty }} {A_{n} \left( {z - ih} \right)^{{ - n}} } . \\  \end{aligned} $$

According to the condition that the magnetic potential cannot be infinite at the horizontal boundary, we put $z = x + ih,\bar{z} = x - ih$ in the magnetic potential additional function, then:

27 $$ f^{r}  = \sum\limits_{{n = 1}}^{\infty } {A_{n} x^{{ - n}} } . $$

In Eq. (27), when $x = 0$, its value cannot be infinite, then for any n, there is $A_{n}  = {\text{0}}$. Therefore, the magnetic potential corresponding to the reflected wave can be expressed as:

28 $$ \phi ^{r}  = \frac{{h_{{15}} }}{{\mu _{{11}} }}w^{r} . $$

According to the derivation process of the magnetic potential corresponding to the reflected wave in the semi-space rare earth giant magnetostrictive medium, the corresponding magnetic potential of the reflected wave in the semi-space rare earth giant magnetostrictive medium containing circular cavity can be expressed as Eq. (28).

### Scattered wave

In complex plane $\left( {z,\bar{z}} \right)$, the scattered wave $w^{s}$ in semi-space with the circular cavity can be constructed by using the symmetric property of the scattered wave and the multipolar coordinate method. The scattered wave are required to satisfy the stress free and magnetic insulation conditions at semi-space boundary $\Gamma _{H}$. Then, the scattered displacement field $w^{s}$ and the magnetic potential corresponding to the scattered wave $\phi ^{s}$ generated by the circular cavity can be expressed as follows:

29 $$ w^{s}  = \sum\limits_{{n =  - \infty }}^{{ + \infty }} {A_{n} \left\{ \begin{gathered}   H_{n}^{{\left( 1 \right)}} \left( {k\left| z \right|} \right)\left( {\frac{z}{{\left| z \right|}}} \right)^{n}  \hfill \\    + H_{n}^{{\left( 1 \right)}} \left( {k\left| {z - ih} \right|} \right)\left( {\frac{{z - 2ih}}{{\left| {z - 2ih} \right|}}} \right)^{{ - n}}  \hfill \\  \end{gathered}  \right\}} , $$

30 $$ \phi ^{s}  = \frac{{h_{{15}} }}{{\mu _{{11}} }}\left( {w^{s}  + f^{s} } \right), $$

where

31 $$ f^{s}  = \sum\limits_{{n = {\text{1}}}}^{{ + \infty }} {\left\{ {B_{n} \left[ {z^{{ - n}}  + \left( {\bar{z} + {\text{2}}ih} \right)^{{ - n}} } \right] + C_{n} \left[ {\bar{z}^{{ - n}}  + \left( {z - {\text{2}}ih} \right)^{{ - n}} } \right]} \right\}} . $$

In order to verify that Eqs. (29) and (30) satisfy the conditions of no stress and magnetic insulation on the horizontal boundary of the semi-space rare earth giant magnetostrictive medium, substituted Eqs. (29) and (30) into the constitutive Eq. (9), then the expressions of the stress and magnetic induction intensity component can be obtained. By substituting the coordinate value $z = x + ih,\bar{z} = x - ih$ at the horizontal boundary, it can be verified that $\tau _{{yz}}  = 0,B_{y}  = 0$.

### Magnetic potential in circular cavity

We know that there is no displacement inside the circular cavity, but it can produce a magnetic field, and the magnetic potential can not be infinite, then the expression of the magnetic potential function can be expressed as follow:

32 $$ \phi ^{c}  = \frac{{h_{{15}} }}{{\mu _{{11}} }}f^{c} . $$

In Eq. (32), $f^{c}$ is the magnetic field additional function inside the circular cavity, which satisfying the Laplace equation in Eq. (4), namely:

33 $$ \frac{{\partial ^{2} f^{c} }}{{\partial z\partial \bar{z}}} = {\text{0}}{\text{.}} $$

Then the magnetic potential $\phi ^{c}$ can be solved as follow:

34 $$ \phi ^{c}  = \frac{{h_{{15}} }}{{\mu _{{11}} }}\left( {\sum\limits_{{n = 0}}^{{ + \infty }} {D_{n} } z^{n}  + \sum\limits_{{n = 0}}^{{ + \infty }} {E_{n} } \bar{z}^{n} } \right), $$

where $D_{n} ,E_{n}$ are the undetermined coefficients.

### Boundary equation

In the semi-space rare earth giant magnetostrictive medium, the total displacement field and the total magnetic potential can be expressed as follows:

35 $$ w^{t}  = w^{i}  + w^{r}  + w^{s} , $$

36 $$ \phi ^{t}  = \phi ^{i}  + \phi ^{r}  + \phi ^{s} . $$

By substituting Eqs. (35) and (36) into the constitutive Eq. (10) to obtain the total stress and the total magnetic induction intensity. Then substituting the total stress, the total magnetic induction intensity and the total magnetic potential into the boundary conditions $\tau _{{rz}}  = 0,B_{r}  = B_{r}^{c} ,\phi ^{t}  = \phi ^{c}$ of the circular cavity, the equations to determine the undetermined coefficients $A_{n} ,B_{n} ,C_{n} ,D_{n} ,E_{n}$ can be obtained as follows:

37 $$ \begin{aligned}    & \sum\limits_{{n =  - \infty }}^{\infty } {A_{n} \xi _{n}^{{(11)}} }  + \sum\limits_{{n = {\text{1}}}}^{\infty } {B_{n} \xi _{n}^{{(12)}} }  + \sum\limits_{{n = {\text{1}}}}^{\infty } {C_{n} \xi _{n}^{{(13)}} }  = \zeta ^{{(1)}}  \\     & \sum\limits_{{n = 1}}^{\infty } {B_{n} \xi _{n}^{{(2{\text{2}})}} }  + \sum\limits_{{n = 1}}^{\infty } {C_{n} \xi _{n}^{{(2{\text{3}})}} }  + \sum\limits_{{n = {\text{0}}}}^{\infty } {D_{n} \xi _{n}^{{(2{\text{4}})}} }  + \sum\limits_{{n = 0}}^{\infty } {E_{n} \xi _{n}^{{(2{\text{5}})}} }  = \zeta ^{{(2)}}  \\     & \sum\limits_{{n =  - \infty }}^{\infty } {A_{n} \xi _{n}^{{(31)}} }  + \sum\limits_{{n = 1}}^{\infty } {B_{n} \xi _{n}^{{(32)}} }  + \sum\limits_{{n = 1}}^{\infty } {C_{n} \xi _{n}^{{(33)}} }  + \sum\limits_{{n = 0}}^{\infty } {D_{n} \xi _{n}^{{(34)}} }  + \sum\limits_{{n = 0}}^{\infty } {E_{n} \xi _{n}^{{(35)}} }  = \zeta ^{{(3)}} , \\  \end{aligned} $$

where

$$ \xi _{n}^{{(11)}}  = \frac{k}{2}c_{{44}} (1 + \lambda )\left\{ \begin{gathered}   \left[ {H_{{n - 1}}^{{(1)}} (k\left| z \right|)\left[ {\frac{z}{{\left| z \right|}}} \right]^{{n - 1}}  - H_{{n + 1}}^{{(1)}} (k\left| {z - 2ih} \right|)\left[ {\frac{{z - 2ih}}{{\left| {z - 2ih} \right|}}} \right]^{{ - (n + 1)}} } \right] \cdot e^{{i\theta }}  \hfill \\    + \left[ { - H_{{n + 1}}^{{(1)}} (k\left| z \right|)\left[ {\frac{z}{{\left| z \right|}}} \right]^{{n + 1}}  + H_{{n - 1}}^{{(1)}} (k\left| {z - 2ih} \right|)\left[ {\frac{{z - 2ih}}{{\left| {z - 2ih} \right|}}} \right]^{{ - (n - 1)}} } \right] \cdot e^{{ - i\theta }}  \hfill \\  \end{gathered}  \right\}, $$

$$ \xi _{n}^{{(12)}}  = \frac{{h_{{15}}^{2} }}{{\mu _{{11}} }}\left\{ {\left[ {( - n)z^{{ - (n + 1)}} } \right]e^{{i\theta }}  + \left[ {( - n)(\bar{z} + 2ih)^{{ - (n + 1)}} } \right] \cdot e^{{ - i\theta }} } \right\}, $$

$$ \xi _{n}^{{({\text{13}})}}  = \frac{{h_{{{\text{15}}}}^{{\text{2}}} }}{{\mu _{{{\text{11}}}} }}\left\{ {\left[ {( - n)(z - {\text{2}}ih)^{{ - (n + 1)}} } \right]e^{{i\theta }}  + \left[ {( - n)\bar{z}^{{ - (n + 1)}} } \right]e^{{ - i\theta }} } \right\}, $$

$$ \xi _{n}^{{({\text{22}})}}  = h_{{15}} \left\{ {\left[ {( - n)z^{{ - (n + 1)}} } \right]e^{{i\theta }}  + \left[ {( - n)(\bar{z} + 2ih)^{{ - (n + 1)}} } \right]e^{{ - i\theta }} } \right\}, $$

$$ \xi _{n}^{{({\text{23}})}}  = h_{{15}} \left\{ {\left[ {\left( { - n} \right)(z - {\text{2}}ih)^{{ - (n + 1)}} } \right] \cdot e^{{i\theta }}  + \left[ {\left( { - n} \right)\bar{z}^{{ - (n + 1)}} } \right] \cdot e^{{ - i\theta }} } \right\}, $$

$$ \xi _{n}^{{({\text{24}})}}  =  - h_{{15}}^{c} nz^{{n - 1}} e^{{i\theta }} , $$

$$ \xi _{n}^{{({\text{25}})}}  =  - h_{{15}}^{c} n\bar{z}^{{n - 1}} e^{{ - i\theta }} , $$

$$ \xi _{n}^{{({\text{31}})}}  = \frac{{h_{{15}} }}{{\mu _{{11}} }}\sum\limits_{{j = 1}}^{{\text{2}}} {S_{n}^{{(j)}} } , $$

$$ \xi _{n}^{{({\text{32}})}}  = \frac{{h_{{15}} }}{{\mu _{{11}} }}\left[ {z^{{ - n}}  + (\bar{z} + 2ih)^{{ - n}} } \right], $$

$$ \xi _{n}^{{({\text{33}})}}  = \frac{{h_{{15}} }}{{\mu _{{11}} }}\left[ {\bar{z}^{{ - n}}  + (z - 2ih)^{{ - n}} } \right], $$

$$ \xi _{n}^{{({\text{34}})}}  = \frac{{h_{{15}} }}{{\mu _{{11}} }}\left( { - z^{n} } \right), $$

$$ \xi _{n}^{{({\text{35}})}}  = \frac{{h_{{15}} }}{{\mu _{{11}} }}\left( { - \bar{z}^{n} } \right), $$

$$ \begin{aligned}    & \zeta ^{{(1)}}  =  - {\text{i}}c_{{44}} (1 + \lambda )kw_{0} \left[ {T_{1} \cos (\theta  - \alpha _{0} ) + T_{2} \cos (\theta  + \alpha _{0} )} \right] \\     & \zeta ^{{(2)}}  = 0 \\     & \zeta ^{{(3)}}  =  - \frac{{h_{{15}} }}{{\mu _{{11}} }} \cdot w_{0} \left[ {T_{1}  + T_{2} } \right]. \\  \end{aligned} $$

In the above equations, $\lambda  = {{h_{{15}}^{2} } \mathord{\left/ {\vphantom {{h_{{15}}^{2} } {c_{{44}} \mu _{{11}} }}} \right. \kern-\nulldelimiterspace} {c_{{44}} \mu _{{11}} }}$ represents the dimensionless comprehensive piezomagnetic parameter, and $h_{{15}}^{c}$ represents the piezomagnetic coefficient in the circular cavity.

By using Fourier expansion method, multiply both sides of Eq. (37) by $e^{{ - im\theta }}$, and integrate on $\left( { - \pi ,\pi } \right)$, then the infinite algebraic equations with coefficients $A_{n}$,$B_{n}$,$C_{n}$,$D_{n}$,$E_{n}$ are obtained. By truncating the finite term, the integrals can be solved. The infinite algebraic equations can be expressed as follows:

38 $$ \begin{aligned}    & \sum\limits_{{n =  - \infty }}^{\infty } {A_{n} \xi _{{mn}}^{{(11)}} }  + \sum\limits_{{n = {\text{1}}}}^{\infty } {B_{n} \xi _{{mn}}^{{(12)}} }  + \sum\limits_{{n = {\text{1}}}}^{\infty } {C_{n} \xi _{{mn}}^{{(13)}} }  = \zeta _{m} ^{{(1)}}  \\     & \sum\limits_{{n = 1}}^{\infty } {B_{n} \xi _{{mn}}^{{(2{\text{2}})}} }  + \sum\limits_{{n = 1}}^{\infty } {C_{n} \xi _{{mn}}^{{(2{\text{3}})}} }  + \sum\limits_{{n = {\text{0}}}}^{\infty } {D_{n} \xi _{{mn}}^{{(2{\text{4}})}} }  + \sum\limits_{{n = 0}}^{\infty } {E_{n} \xi _{{mn}}^{{(2{\text{5}})}} }  = \zeta _{m} ^{{(2)}}  \\     & \sum\limits_{{n =  - \infty }}^{\infty } {A_{n} \xi _{{mn}}^{{(31)}} }  + \sum\limits_{{n = 1}}^{\infty } {B_{n} \xi _{{mn}}^{{(32)}} }  + \sum\limits_{{n = 1}}^{\infty } {C_{n} \xi _{{mn}}^{{(33)}} }  + \sum\limits_{{n = 0}}^{\infty } {E_{n} \xi _{{mn}}^{{(34)}} }  + \sum\limits_{{n = 0}}^{\infty } {E_{n} \xi _{{mn}}^{{(35)}} }  = \zeta _{m} ^{{(3)}} . \\  \end{aligned} $$

Among them $\xi _{{mn}}^{{(11)}}  = \frac{1}{{2\pi }}\int_{{ - \pi }}^{\pi } {\xi _{n}^{{{\text{(11)}}}} } e^{{ - {\text{i}}m\theta }} d\theta ,\zeta _{m}^{{(1)}}  = \frac{1}{{2\pi }}\int_{{ - \pi }}^{\pi } {\zeta ^{{(1)}} } e^{{ - {\text{i}}m\theta }} d\theta$, and the rest are all the same. The undetermined coefficients $A_{n}$,$B_{n}$,$C_{n}$,$D_{n}$,$E_{n}$ can be determined by solving the infinite algebraic Eq. (38).

## Dynamic stress concentration factor and magnetic field intensity concentration factor

The total stress and the total magnetic field strength around the circular cavity in the semi-space rare earth giant magnetostrictive medium can be expressed as follows:

39 $$ \begin{aligned}    & \tau _{{\theta z}}^{t}  = \tau _{{\theta z}}^{i}  + \tau _{{\theta z}}^{r}  + \tau _{{\theta z}}^{s}  \\     & H_{\theta }^{t}  =  - i\left( {\frac{{\partial \phi }}{{\partial z}}e^{{i\theta }}  - \frac{{\partial \phi }}{{\partial \bar{z}}}e^{{ - i\theta }} } \right), \\  \end{aligned} $$

where

$$ \begin{aligned}   \tau _{{\theta z}}^{t}  &  =  - ic_{{44}} (1 + \lambda )kw_{0} \left[ {T_{1} \sin (\theta  - \alpha _{0} ) + T_{{\text{2}}} \sin (\theta  + \alpha _{0} )} \right] \\     & \quad  + \frac{{ik(1 + \lambda )c_{{44}} }}{2}\sum\limits_{{n =  - \infty }}^{\infty } {A_{n} } \left\{ \begin{gathered}   \left[ {H_{{n - 1}}^{{(1)}} (k\left| z \right|)\left[ {\frac{z}{{\left| z \right|}}} \right]^{{n - 1}}  - H_{{n + 1}}^{{(1)}} (k\left| {z - 2ih} \right|)\left[ {\frac{{z - 2ih}}{{\left| {z - 2ih} \right|}}} \right]^{{ - (n + 1)}} } \right]e^{{i\theta }}  \hfill \\    - \left[ { - H_{{n + 1}}^{{(1)}} (k\left| z \right|)\left[ {\frac{z}{{\left| z \right|}}} \right]^{{n + 1}}  + H_{{n - 1}}^{{(1)}} (k\left| {z - 2ih} \right|)\left[ {\frac{{z - 2ih}}{{\left| {z - 2ih} \right|}}} \right]^{{ - (n - 1)}} } \right]e^{{ - i\theta }}  \hfill \\  \end{gathered}  \right\} \\     & \quad  + i\frac{{h_{{{\text{15}}}}^{{\text{2}}} }}{{\mu _{{{\text{11}}}} }}\sum\limits_{{n = 1}}^{\infty } {B_{n} } \left\{ \begin{gathered}   \left[ {\left( { - n} \right)z^{{ - (n + 1)}} } \right]e^{{i\theta }}  \hfill \\    - \left[ {\left( { - n} \right)(\bar{z} + 2ih)^{{ - (n + 1)}} } \right]e^{{ - i\theta }}  \hfill \\  \end{gathered}  \right\} \\     & \quad  + i\frac{{h_{{{\text{15}}}}^{{\text{2}}} }}{{\mu _{{{\text{11}}}} }}\sum\limits_{{n = 1}}^{\infty } {C_{n} } \left\{ \begin{gathered}   \left[ {\left( { - n} \right)(z - {\text{2}}ih)^{{ - (n + 1)}} } \right]e^{{i\theta }}  \hfill \\    - \left[ {\left( { - n} \right)\bar{z}^{{ - (n + 1)}} } \right]e^{{ - i\theta }}  \hfill \\  \end{gathered}  \right\}, \\  \end{aligned} $$

$$ \begin{aligned}   H_{\theta }^{t}  &  =  - i\left( {\frac{{\partial \phi }}{{\partial z}}e^{{i\theta }}  - \frac{{\partial \phi }}{{\partial \bar{z}}}e^{{ - i\theta }} } \right) \\     &  = \frac{{h_{{15}} }}{{\mu _{{11}} }}ikw_{0} \left[ {T_{1} \sin (\theta  - \alpha _{0} ) + T_{{\text{2}}} \sin (\theta  + \alpha _{0} )} \right] \\     & \quad  - \frac{{ikh_{{15}} }}{{2\mu _{{11}} }}\sum\limits_{{n =  - \infty }}^{\infty } {A_{n} } \left\{ \begin{gathered}   \left[ {H_{{n - 1}}^{{(1)}} (k\left| z \right|)\left[ {\frac{z}{{\left| z \right|}}} \right]^{{n - 1}}  - H_{{n + 1}}^{{(1)}} (k\left| {z - 2ih} \right|)\left[ {\frac{{z - 2ih}}{{\left| {z - 2ih} \right|}}} \right]^{{ - (n + 1)}} } \right]e^{{i\theta }}  \hfill \\    - \left[ { - H_{{n + 1}}^{{(1)}} (k\left| z \right|)\left[ {\frac{z}{{\left| z \right|}}} \right]^{{n + 1}}  + H_{{n - 1}}^{{(1)}} (k\left| {z - 2ih} \right|)\left[ {\frac{{z - 2ih}}{{\left| {z - 2ih} \right|}}} \right]^{{ - (n - 1)}} } \right]e^{{ - i\theta }}  \hfill \\  \end{gathered}  \right\} \\     & \quad  - i\frac{{h_{{{\text{15}}}} }}{{\mu _{{{\text{11}}}} }}\sum\limits_{{n = 1}}^{\infty } {B_{n} } \left\{ \begin{gathered}   \left[ {\left( { - n} \right)z^{{ - (n + 1)}} } \right]e^{{i\theta }}  \hfill \\    - \left[ {\left( { - n} \right)(\bar{z} + 2{\text{i}}h)^{{ - (n + 1)}} } \right]e^{{ - i\theta }}  \hfill \\  \end{gathered}  \right\} \\     & \quad  - i\frac{{h_{{{\text{15}}}} }}{{\mu _{{{\text{11}}}} }}\sum\limits_{{n = 1}}^{\infty } {C_{n} } \left\{ \begin{gathered}   \left[ {\left( { - n} \right)(z - {\text{2i}}h)^{{ - (n + 1)}} } \right]e^{{i\theta }}  \hfill \\    - \left[ {\left( { - n} \right)\bar{z}^{{ - (n + 1)}} } \right]e^{{ - i\theta }}  \hfill \\  \end{gathered}  \right\}. \\  \end{aligned} $$

Then the dynamic stress concentration factor of the circular cavity edge can be expressed as follow:

40 $$ \tau _{{\theta z}}^{*}  = \left| {\frac{{\left. {\tau _{{\theta z}}^{t} } \right|_{{r = R}} }}{{\tau _{0} }}} \right|, $$

where $\tau _{{\text{0}}}  = c_{{44}}\; kw_{0} ({\text{1}} + \lambda )$ represents the maximum value of incident stress.

The magnetic field intensity concentration factor of the circular cavity edge can be expressed as follow:

41 $$ H_{\theta }^{{\text{*}}}  = \left| {\frac{{\left. {H_{\theta }^{t} } \right|_{{r = R}} }}{{H_{0} }}} \right|, $$

where $H_{0}  = \frac{{h_{{15}} }}{{\mu _{{11}} }}\;kw_{0}$ represents the intensity amplitude of circumferential magnetic field corresponding to the incident wave.

## Numerical calculation and analysis

Since we need to truncate finite terms to solve the infinite algebraic equations, the selection of truncated terms will affect the final calculation results. Because there is no displacement field inside the circular cavity, the radial stress at the boundary of the circular cavity should be 0. The accuracy of the analysis method can be tested by the residual dimensionless stress at the boundary of the circular cavity. The results show that when the maximum residual stress is less than 5%, the accuracy of the calculated results can be guaranteed. In this paper, truncation of Fourier series is taken $m = n = {\text{15}}$, which is sufficient to make the dimensionless stress residual reach $10^{{ - 3}}$.

**Figure 2:**
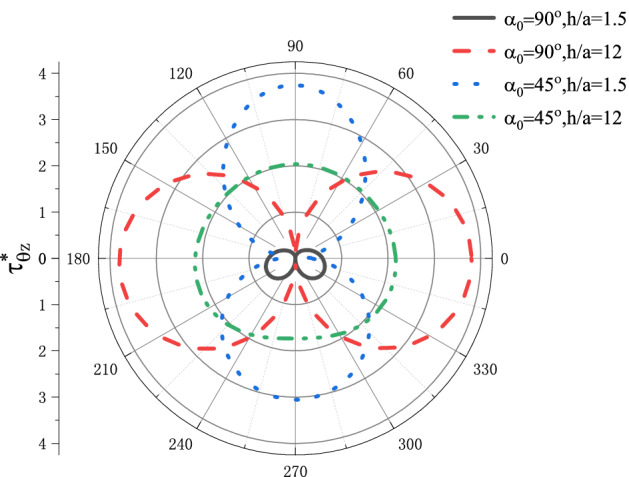
Verification of the present method (compared with reference^22^).

In this paper, the calculation result diagrams of dynamic stress concentration factor (DSCF) and magnetic field intensity concentration factor (MFICF) around the circular cavity are presented, which vary with the dimensionless wave number $kR$, dimensionless piezomagnetic parameters $\lambda$, material geometrical parameters, incident angle $\alpha _{{\text{0}}}$ and the geometric position of the circular cavity ${h \mathord{\left/ {\vphantom {h R}} \right. \kern-\nulldelimiterspace} R}$. When $\lambda  = {\text{0}},h_{{15}}  = 0,k_{{11}}  = 0,h_{{15}}^{c}  = 0$, the model in this paper degenerates into the elastic medium with a circular cavity in semi-space. The same parameters in reference^22^ have been used to solve the distribution of $\tau _{{\theta z}}^{*}$, as shown in Fig. 2. The comparison shows that the calculated results are in good agreement with the results in the references. Therefore, the calculation method in this paper is feasible.

**Figure 3:**
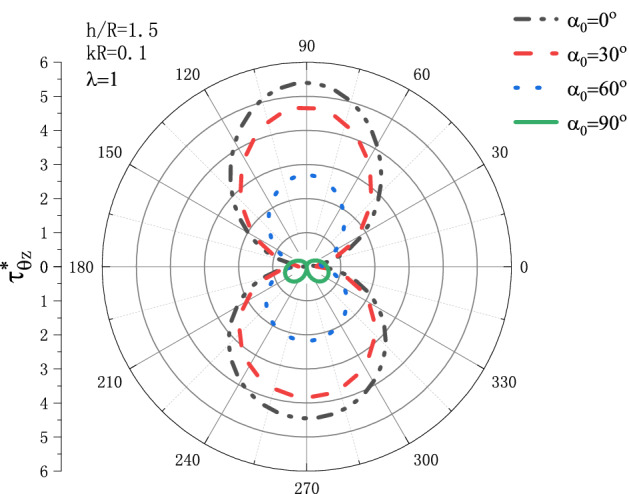
Variation of $\tau _{{\theta z}}^{*}$ vs. incident angle $\alpha _{{\text{0}}}$ of the SH wave when ${h \mathord{\left/ {\vphantom {h R}} \right. \kern-\nulldelimiterspace} R} = 1.5$

**Figure 4:**
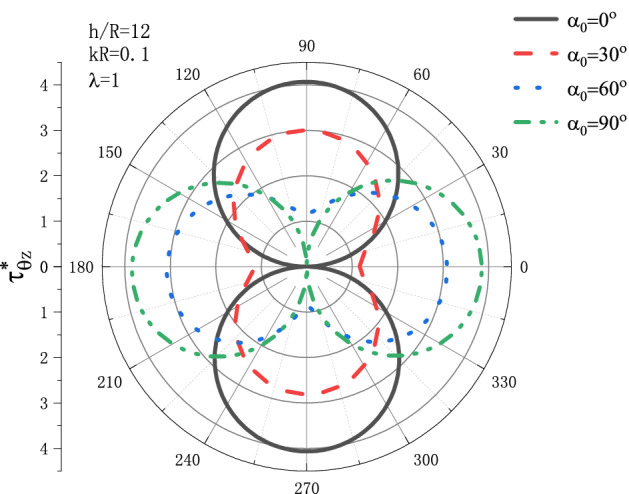
Variation of $\tau _{{\theta z}}^{*}$ vs. incident angle $\alpha _{{\text{0}}}$ of the SH wave when ${h \mathord{\left/ {\vphantom {h R}} \right. \kern-\nulldelimiterspace} R} = 1{\text{2}}$.

Figures 3 and 4 show the distribution situation of the DSCF $\tau _{{\theta z}}^{*}$ around the circular cavity when the SH wave is incident at different angles $\alpha _{{\text{0}}}$. Figure 3 is the distribution of $\tau _{{\theta z}}^{*}$ when ${h \mathord{\left/ {\vphantom {h R}} \right. \kern-\nulldelimiterspace} R} = 1.5$ and Fig. 4 is the distribution of $\tau _{{\theta z}}^{*}$ when ${h \mathord{\left/ {\vphantom {h R}} \right. \kern-\nulldelimiterspace} R} = {\text{12}}$. It can be seen that when ${h \mathord{\left/ {\vphantom {h R}} \right. \kern-\nulldelimiterspace} R} = 1.5$, that is, when the circular cavity is close to the upper boundary, the value of $\tau _{{\theta z}}^{*}$ near the circular cavity reaches its maximum value at position $\theta  = {\pi  \mathord{\left/ {\vphantom {\pi  {\text{2}}}} \right. \kern-\nulldelimiterspace} {\text{2}}}$, and the $\tau _{{\theta z}}^{*}$ decreases as the incident angle of SH wave becomes vertical, that is, the value of $\tau _{{\theta z}}^{*}$ is the largest when SH wave is horizontally incident. When ${h \mathord{\left/ {\vphantom {h R}} \right. \kern-\nulldelimiterspace} R} = {\text{12}}$, that is, when the circular cavity is far from the upper boundary, the DSCF reaches its maximum value at $\theta  = {\pi  \mathord{\left/ {\vphantom {\pi  {\text{2}}}} \right. \kern-\nulldelimiterspace} {\text{2}}}$ as the SH wave is horizontally incident. When SH wave is vertically incident, $\tau _{{\theta z}}^{*}$ reaches its maximum value at position $\theta  = {\text{0}}$. This indicates that $\tau _{{\theta z}}^{*}$ is more significantly affected when the distance between the boundary and the circular cavity is closer.

**Figure 5:**
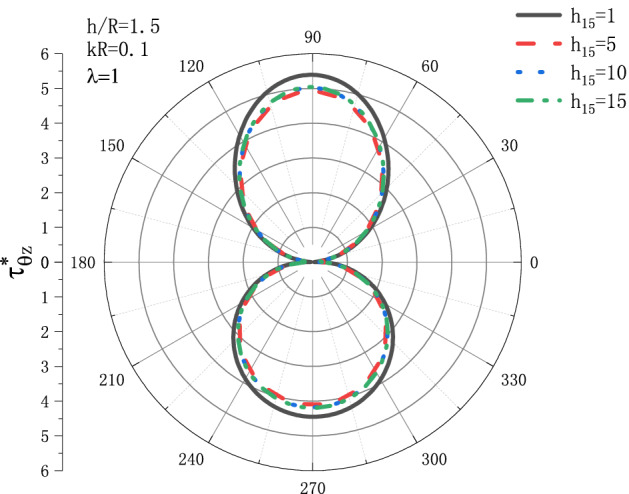
Variation of $\tau _{{\theta z}}^{*}$ vs. the ratio of $h_{{15}}$ when incident frequency $kR = 0.1$

**Figure 6:**
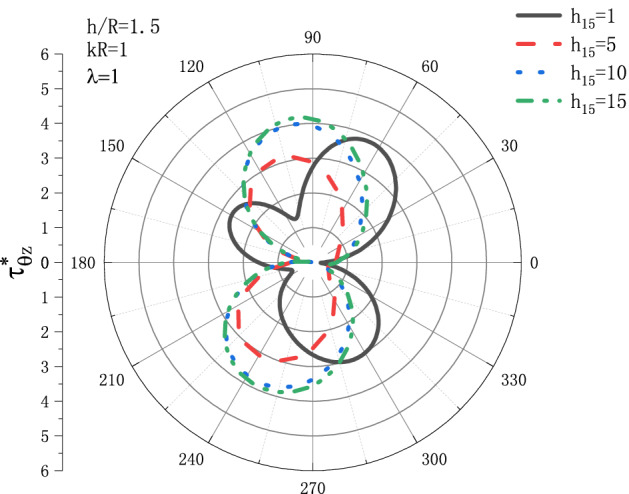
Variation of $\tau _{{\theta z}}^{*}$ vs. the ratio of $h_{{15}}$ when incident frequency $kR = {\text{1}}$.

Figures 5 and 6 show the variation of DSCF $\tau _{{\theta z}}^{*}$ with respect to $h_{{15}}$ under the conditions of ${h \mathord{\left/ {\vphantom {h R}} \right. \kern-\nulldelimiterspace} R} = 1.5,\lambda  = {\text{1}}$ when SH wave incidence is horizontal. Figure 5 is the distribution of $\tau _{{\theta z}}^{*}$ when $kR = 0.1$, and Fig. 6 is the distribution of $\tau _{{\theta z}}^{*}$ when $kR = {\text{1}}$. It can be seen that when $kR = 0.1$, the DSCF $\tau _{{\theta z}}^{*}$ first increases with $h_{{15}}$, then slightly decreases, and then tends to be stable. When $kR = {\text{1}}$, the position where $\tau _{{\theta z}}^{*}$ reaches the maximum value with the increase of $h_{{15}}$ gradually moves from $\theta  = {\pi  \mathord{\left/ {\vphantom {\pi  {\text{3}}}} \right. \kern-\nulldelimiterspace} {\text{3}}}$ to $\theta  = {\pi  \mathord{\left/ {\vphantom {\pi  {\text{2}}}} \right. \kern-\nulldelimiterspace} {\text{2}}}$, and $\tau _{{\theta z}}^{*}$ tends to a fixed value when $h_{{15}}$ reaches 10.

**Figure 7:**
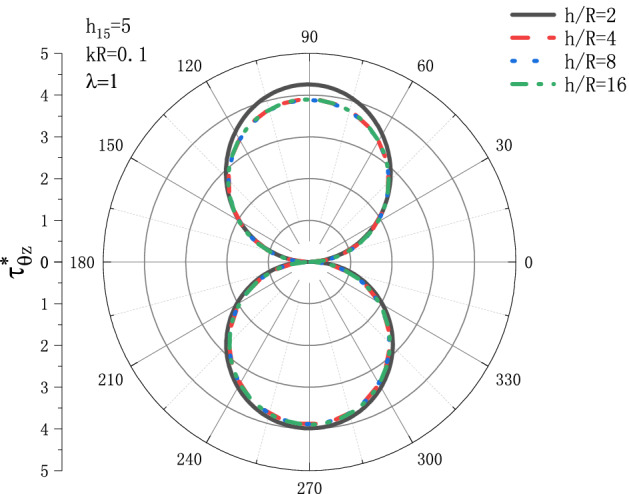
Variation of $\tau _{{\theta z}}^{*}$ vs. the ratio of ${h \mathord{\left/ {\vphantom {h R}} \right. \kern-\nulldelimiterspace} R}$ when incident frequency $kR = {\text{0}}{\text{.1}}$.

**Figure 8:**
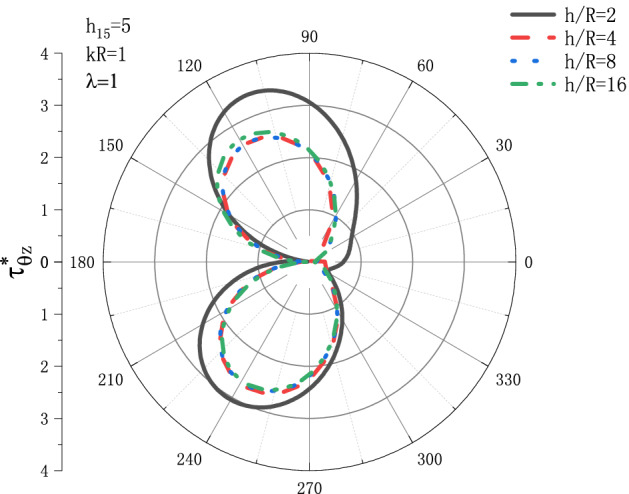
Variation of $\tau _{{\theta z}}^{*}$ vs. the ratio of ${h \mathord{\left/ {\vphantom {h R}} \right. \kern-\nulldelimiterspace} R}$ when incident frequency $kR = {\text{1}}$.

Figures 7 and 8 show the variation of dynamic stress coefficient factor $\tau _{{\theta z}}^{*}$ with respect to ${h \mathord{\left/ {\vphantom {h R}} \right. \kern-\nulldelimiterspace} R}$ under the conditions of $h_{{15}}  = 5,\lambda  = 1$ when SH wave incidence is horizontal. Figure 7 shows the distribution of the dynamic stress coefficient factor around the circular cavity at the incident frequency $kR = 0.1$, and Fig. 8 shows the distribution of the dynamic stress coefficient factor around the circular cavity at the incident frequency $kR = {\text{1}}$. It can be seen from Fig. 7 that when incident frequency is $kR = 0.1$, $\tau _{{\theta z}}^{*}$ decreases slightly with the increase of ${h \mathord{\left/ {\vphantom {h R}} \right. \kern-\nulldelimiterspace} R}$. When the value of ${h \mathord{\left/ {\vphantom {h R}} \right. \kern-\nulldelimiterspace} R}$ reaches 4, the value of $\tau _{{\theta z}}^{*}$ almost no longer changes, and finally stabilizes at about $\tau _{{\theta z}}^{*}  = {\text{3}}{\text{.8}}$. The position where the DSCF reaches the maximum is $\theta  = {\pi  \mathord{\left/ {\vphantom {\pi  {\text{2}}}} \right. \kern-\nulldelimiterspace} {\text{2}}}$. It can be seen from Fig. 8, in the case of incident frequency $kR = {\text{1}}$, the variation trend of $\tau _{{\theta z}}^{*}$ with the increase of ${h \mathord{\left/ {\vphantom {h R}} \right. \kern-\nulldelimiterspace} R}$ is the same as that of Fig. 7, and $\tau _{{\theta z}}^{*}$ decreases slightly with the increase of ${h \mathord{\left/ {\vphantom {h R}} \right. \kern-\nulldelimiterspace} R}$. When the value of ${h \mathord{\left/ {\vphantom {h R}} \right. \kern-\nulldelimiterspace} R}$ reaches 4, the value of $\tau _{{\theta z}}^{*}$ almost no longer changes, and the final stability is about $\tau _{{\theta z}}^{*}  = {\text{2}}{\text{.7}}$, but the position where the DSCF $\tau _{{\theta z}}^{*}$ reaches the maximum is $\theta  = {{{\text{2}}\pi } \mathord{\left/ {\vphantom {{{\text{2}}\pi } {\text{3}}}} \right. \kern-\nulldelimiterspace} {\text{3}}}$. Compared with Figs. 7 and 8, the DSCF $\tau _{{\theta z}}^{*}$ tends to decrease when the incident frequency $kR$ increases.

**Figure 9:**
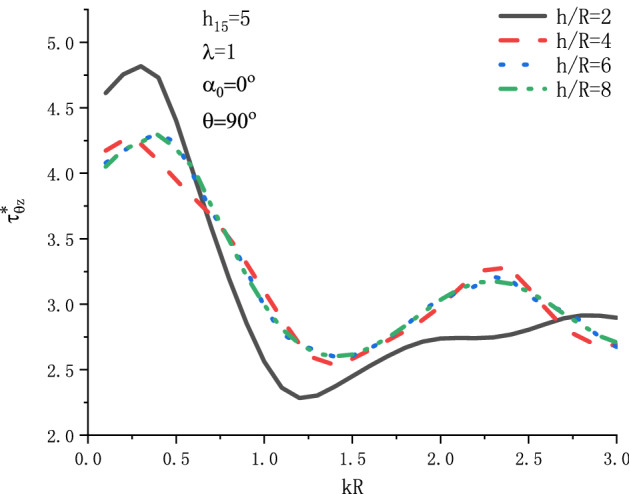
Variation of $\tau _{{\theta z}}^{*}$ vs. $kR$ when SH wave incident horizontally.

**Figure 10:**
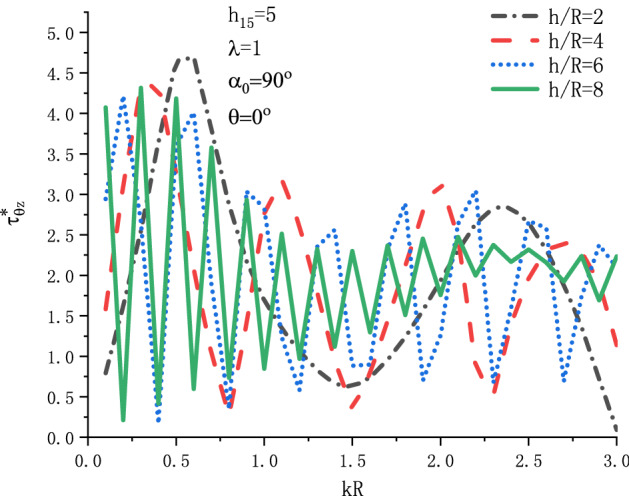
Variation of $\tau _{{\theta z}}^{*}$ vs. $kR$ when SH wave incident vertically.

Figure 9 shows the variation of $\tau _{{\theta z}}^{*}$ at position $\theta  = {\pi  \mathord{\left/ {\vphantom {\pi  {\text{2}}}} \right. \kern-\nulldelimiterspace} {\text{2}}}$ with respect to the incident wave number $kR$ when SH wave is incident horizontally under different ${h \mathord{\left/ {\vphantom {h R}} \right. \kern-\nulldelimiterspace} R}$. Figure 10 shows the variation of $\tau _{{\theta z}}^{*}$ at position $\theta  = {\pi  \mathord{\left/ {\vphantom {\pi  {\text{2}}}} \right. \kern-\nulldelimiterspace} {\text{2}}}$ with respect to the incident wave number $kR$ when SH wave is incident vertically under different ${h \mathord{\left/ {\vphantom {h R}} \right. \kern-\nulldelimiterspace} R}$. It can be seen from Fig. 9 that when SH wave is horizontally incident, the DSCF $\tau _{{\theta z}}^{*}$ will have a maximum value within the range of $kR = 0.2\sim 0.4$. Taking ${h \mathord{\left/ {\vphantom {h R}} \right. \kern-\nulldelimiterspace} R} = 2$, the maximum value of $\tau _{{\theta z}}^{*}$ is about 4.8. It can be seen from Fig. 10 that when SH wave is incident vertically, the fluctuation of the distribution curve of DSCF around the circular cavity is intensified, which reflects that the horizontal boundary has a greater impact.

**Figure 11:**
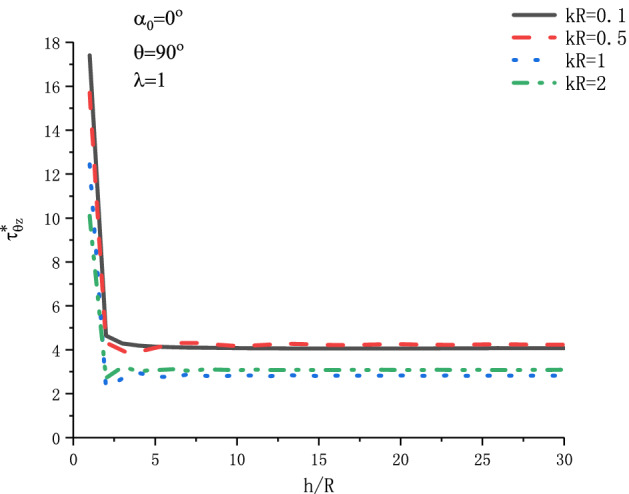
Variation of $\tau _{{\theta z}}^{*}$ vs. ${h \mathord{\left/ {\vphantom {h R}} \right. \kern-\nulldelimiterspace} R}$ when SH wave incident horizontally.

**Figure 12:**
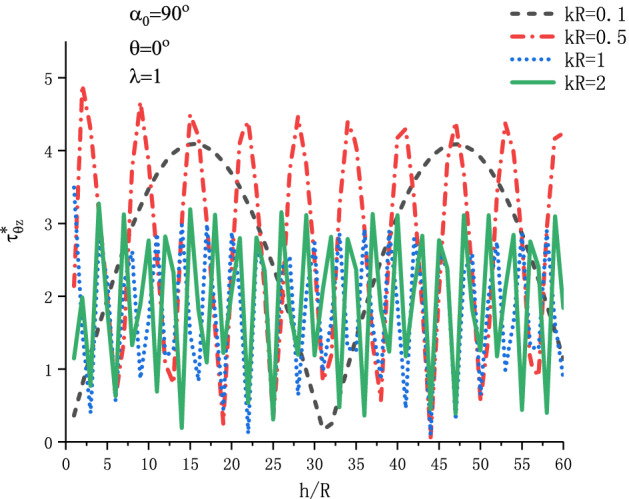
Variation of $\tau _{{\theta z}}^{*}$ vs. ${h \mathord{\left/ {\vphantom {h R}} \right. \kern-\nulldelimiterspace} R}$ when SH wave incident vertically.

Figure 11 shows the variation of $\tau _{{\theta z}}^{*}$ at position $\theta  = {\pi  \mathord{\left/ {\vphantom {\pi  {\text{2}}}} \right. \kern-\nulldelimiterspace} {\text{2}}}$ with respect to ${h \mathord{\left/ {\vphantom {h R}} \right. \kern-\nulldelimiterspace} R}$ when SH wave is incident horizontally under different incident frequency $kR$. Figure 12 shows the variation of $\tau _{{\theta z}}^{*}$ at position $\theta  = {\text{0}}$ with respect to ${h \mathord{\left/ {\vphantom {h R}} \right. \kern-\nulldelimiterspace} R}$ when SH wave is incident vertically under different incident wave number $kR$. The graph discusses the four values of $kR$ respectively. It can be seen from Fig. 11 that when ${h \mathord{\left/ {\vphantom {h R}} \right. \kern-\nulldelimiterspace} R} = {\text{1}}$, the DSCF reaches its maximum value. With the increase of ${h \mathord{\left/ {\vphantom {h R}} \right. \kern-\nulldelimiterspace} R}$, the DSCF $\tau _{{\theta z}}^{*}$ decreases abruptly, and then oscillates gradually to be stable. It can be seen from Fig. 12 that with the increase of wave number $kR$, the DSCF curve at the edge of the circular cavity changes more strongly.

**Figure 13:**
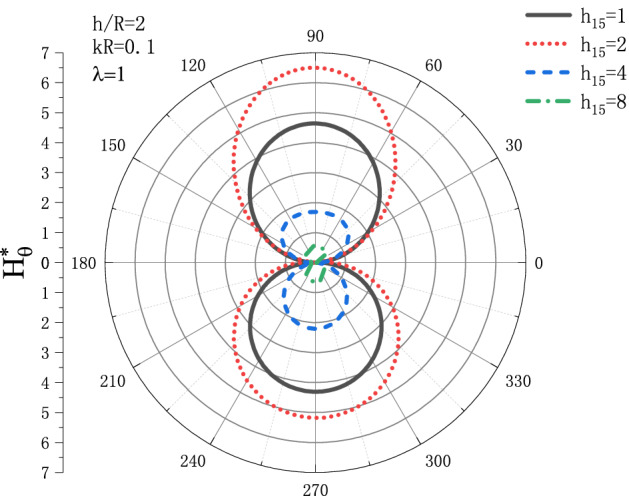
Variation of $H_{\theta }^{{\text{*}}}$ vs. the ratio of $h_{{15}}$ when incident frequency $kR = 0.1$

**Figure 14:**
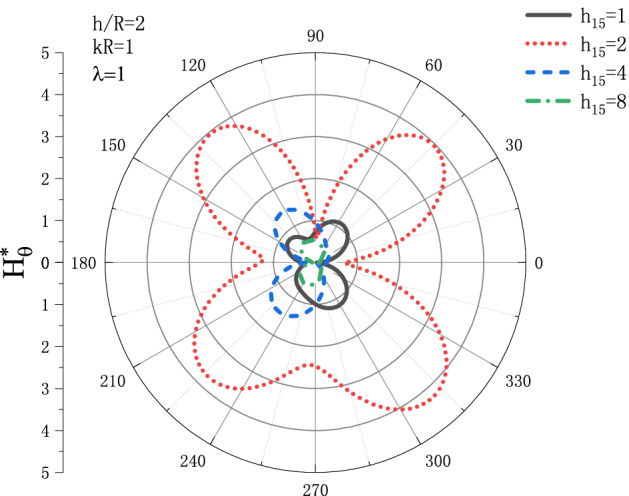
Variation of $H_{\theta }^{{\text{*}}}$ vs. the ratio of $h_{{15}}$ when incident frequency $kR = {\text{1}}$.

Figures 13 and 14 show the distribution of the MFICF $H_{\theta }^{{\text{*}}}$ around the circular cavity varying with the piezomagnetic coefficient $h_{{15}}$ when the steady-state SH wave is horizontally incident, so that let the comprehensive piezomagnetic parameter $\lambda  = {\text{1}}$ and the geometric position of the circular cavity $h/R = {\text{2}}$, the distribution discussed at incident frequency $kR = 0.1$ and $kR = {\text{1}}$. Figure 13 shows the distribution of the MFICF around the circular cavity at the incident frequency $kR = 0.1$, and Fig. 14 shows the distribution of the MFICF around the circular cavity when the incident frequency is $kR = {\text{1}}$. It can be seen from Fig. 13 that when the SH wave is horizontally incident, the $H_{\theta }^{{\text{*}}}$ at the upper and lower points of the circular cavity reaches a maximum of about 6.5. The distribution of $H_{\theta }^{{\text{*}}}$ shows a trend of increasing at first and then decreasing. Take an example, the value of $H_{\theta }^{{\text{*}}}$ at $h_{{15}}  = 2$ is larger than that of $h_{{15}}  = {\text{1}}$, but $H_{\theta }^{{\text{*}}}$ decreases rapidly with the further increase of $h_{{15}}$. It can be seen from Fig. 14 that $H_{\theta }^{{\text{*}}}$ also increases at first and then decreases greatly with the increase of $h_{{15}}$, and the position where $H_{\theta }^{{\text{*}}}$ get the maximum is moved from $\theta  = {\pi  \mathord{\left/ {\vphantom {\pi  {\text{3}}}} \right. \kern-\nulldelimiterspace} {\text{3}}}$ to $\theta  = {\text{2}}{\pi  \mathord{\left/ {\vphantom {\pi  {\text{3}}}} \right. \kern-\nulldelimiterspace} {\text{3}}}$.

**Figure 15:**
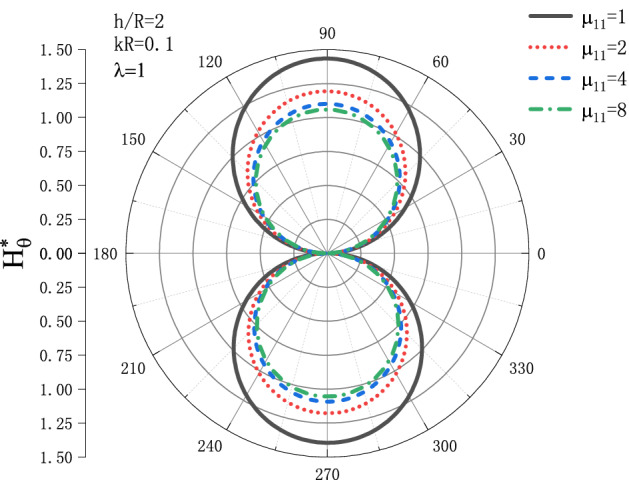
Variation of $H_{\theta }^{{\text{*}}}$ vs. the ratio of $\mu _{{11}}$ when incident frequency $kR = 0.1$

**Figure 16:**
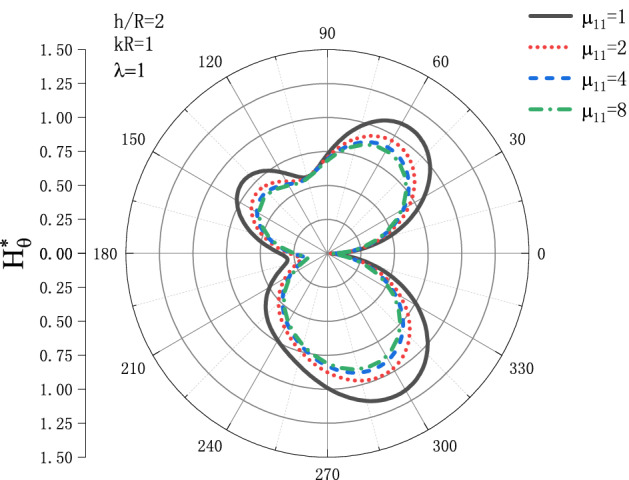
Variation of $H_{\theta }^{{\text{*}}}$ vs. the ratio of $\mu _{{11}}$ when incident frequency $kR = {\text{1}}$.

Figures 15 and 16 show the distribution of the MFICF $H_{\theta }^{{\text{*}}}$ around the circular cavity varying with the permeability $\mu _{{11}}$ when the steady-state SH wave is horizontally incident, so that let the comprehensive piezomagnetic parameter $\lambda  = {\text{1}}$ and the geometric position of the circular cavity $h/R = {\text{2}}$, the distribution discussed at incident frequency $kR = 0.1$ and $kR = {\text{1}}$. Figure 15 shows the distribution of the MFICF around the circular cavity at the incident frequency $kR = 0.1$, and Fig. 16 shows the distribution of the MFICF around the circular cavity when the incident frequency is $kR = {\text{1}}$. It can be seen from Fig. 15 that when the SH wave is horizontally incident, the MFICF at the upper and lower points of the circular cavity reaches a maximum, and $H_{\theta }^{{\text{*}}}$ decreases with the increase of $\mu _{{11}}$. When $\mu _{{11}}$ increases to about 4, $H_{\theta }^{{\text{*}}}$ tends to be stable, and $H_{\theta }^{{\text{*}}}$ no longer decreases with the increase of $\mu _{{11}}$. It can be seen from Fig. 16 that when the SH wave incident horizontally, the MFICF around the circular cavity decreases with the increase of $\mu _{{11}}$, and when about $\mu _{{11}}  = {\text{4}}$, $H_{\theta }^{{\text{*}}}$ tends to be stable. Unlike the quasi-static $kR = 0.1$ of Fig. 15, when the SH wave incident horizontally, $H_{\theta }^{{\text{*}}}$ around the circular cavity defect reaches the maximum at $\theta  = {\pi  \mathord{\left/ {\vphantom {\pi  {\text{3}}}} \right. \kern-\nulldelimiterspace} {\text{3}}}$ and $\theta  = {\text{5}}{\pi  \mathord{\left/ {\vphantom {\pi  {\text{3}}}} \right. \kern-\nulldelimiterspace} {\text{3}}}$.

**Figure 17:**
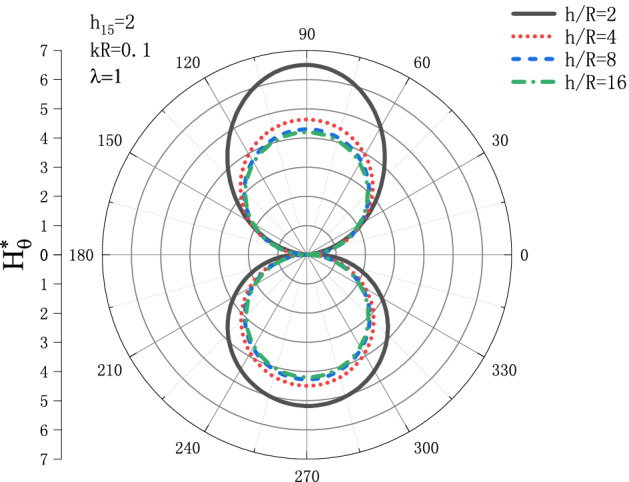
Variation of $H_{\theta }^{{\text{*}}}$ vs. the ratio of ${h \mathord{\left/ {\vphantom {h R}} \right. \kern-\nulldelimiterspace} R}$ when incident frequency $kR = 0.1$

**Figure 18:**
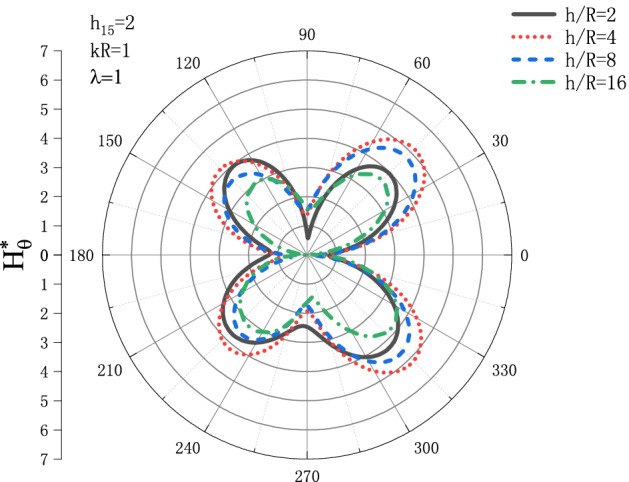
Variation of $H_{\theta }^{{\text{*}}}$ vs. the ratio of ${h \mathord{\left/ {\vphantom {h R}} \right. \kern-\nulldelimiterspace} R}$ when incident frequency $kR = {\text{1}}$.

Figures 17 and 18 show the distribution of the MFICF $H_{\theta }^{{\text{*}}}$ around the circular cavity varying with the geometric position of the circular cavity $h/R$ when the steady-state SH wave is horizontally incident, so that let the comprehensive piezomagnetic parameter $\lambda  = {\text{1}}$ and piezomagnetic coefficient $h_{{15}}  = {\text{2}}$, the distribution discussed at incident frequency $kR = 0.1$ and $kR = {\text{1}}$. Figure 17 shows the distribution of the MFICF around the circular cavity at the incident frequency $kR = 0.1$, and Fig. 18 shows the distribution of the MFICF around the circular cavity when the incident frequency is $kR = {\text{1}}$. It can be seen from Fig. 17 that when the SH wave incident horizontally, the MFICF at the upper and lower points of the circular cavity reaches a maximum. With the increase of $h/R$, the MFICF decreases, and when $h/R$ increases to about 8, the value of $H_{\theta }^{{\text{*}}}$ gradually tends to a fixed value and no longer changes. This reflects that in the quasi-static case $kR = 0.1$, when the distance between the horizontal boundary and the circular cavity reaches a certain value, it will no longer have a great influence on the MFICF around the circular cavity. It can be seen from Fig. 18 that when the SH wave is horizontally incident, the distribution curve of $H_{\theta }^{{\text{*}}}$ shows a butterfly shape, and the positions when $H_{\theta }^{{\text{*}}}$ reaches a maximum at $\theta  = {\pi  \mathord{\left/ {\vphantom {\pi  {\text{4}}}} \right. \kern-\nulldelimiterspace} {\text{4}}}$, $\theta  = {{{\text{3}}\pi } \mathord{\left/ {\vphantom {{{\text{3}}\pi } {\text{4}}}} \right. \kern-\nulldelimiterspace} {\text{4}}}$, $\theta  = {{{\text{5}}\pi } \mathord{\left/ {\vphantom {{{\text{5}}\pi } {\text{4}}}} \right. \kern-\nulldelimiterspace} {\text{4}}}$ and $\theta  = {{{\text{7}}\pi } \mathord{\left/ {\vphantom {{{\text{7}}\pi } {\text{4}}}} \right. \kern-\nulldelimiterspace} {\text{4}}}$. The value of $H_{\theta }^{{\text{*}}}$ increases at first and then decreases with the increase of $h/R$, which shows that when the distance between the circular cavity and the horizontal boundary reaches a certain distance, the influence of the horizontal free surface on the $H_{\theta }^{{\text{*}}}$ becomes smaller.

**Figure 19:**
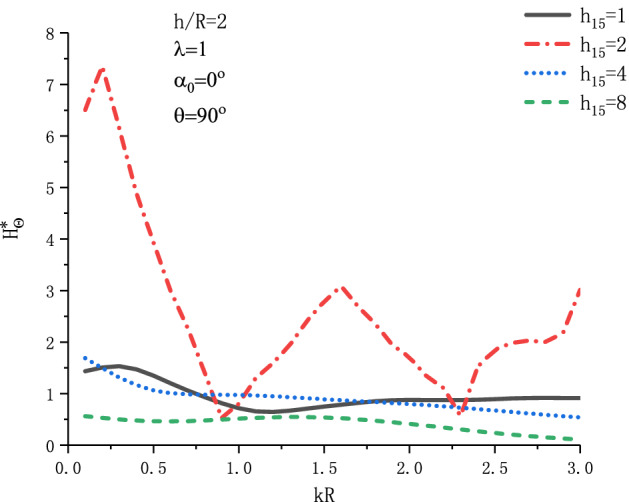
Variation of $H_{\theta }^{{\text{*}}}$ vs. $kR$ when SH wave incident horizontally ($h_{{15}}$).

**Figure 20:**
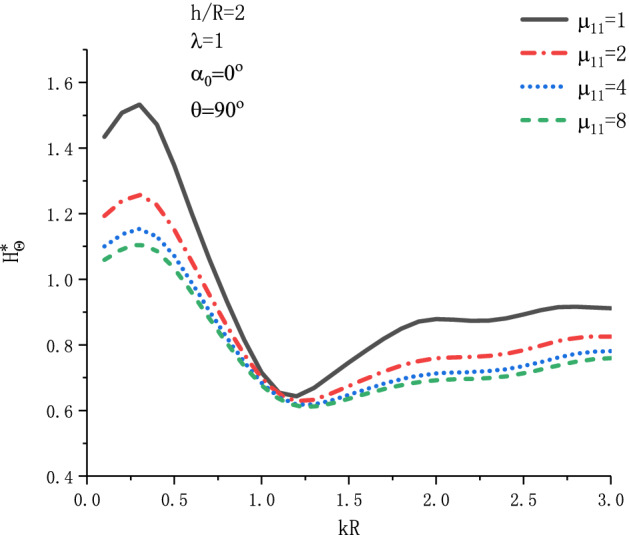
Variation of $H_{\theta }^{{\text{*}}}$ vs. $kR$ when SH wave incident horizontally ($\mu _{{{\text{11}}}}$).

Figure 19 shows the variation of $H_{\theta }^{{\text{*}}}$ at position $\theta  = {\pi  \mathord{\left/ {\vphantom {\pi  {\text{2}}}} \right. \kern-\nulldelimiterspace} {\text{2}}}$ with respect to $kR$ when SH wave is incident horizontally under different $h_{{15}}$. Figure 20 shows the variation of $H_{\theta }^{{\text{*}}}$ at position $\theta  = {\pi  \mathord{\left/ {\vphantom {\pi  {\text{2}}}} \right. \kern-\nulldelimiterspace} {\text{2}}}$ with respect to $kR$ when SH wave is incident horizontally under different $\mu _{{{\text{11}}}}$. As can be seen from Fig. 19, with the increase of $h_{{15}}$, the MFICF presents a trend of first increasing and then decreasing, and with the increase of wave number $kR$, $H_{\theta }^{*}$ presents a trend of oscillation decreasing. It can be seen from Fig. 20, when the SH wave is horizontally incident, $H_{\theta }^{*}$ will have a maximum value in the range of $kR = 0.2\sim 0.4$. Taking $\mu _{{{\text{11}}}}  = {\text{1}}$, the maximum value of $H_{\theta }^{*}$ is about 1.58. With the increase of $\mu _{{{\text{11}}}}$, the MFICF gradually tends to a stable value.

**Figure 21:**
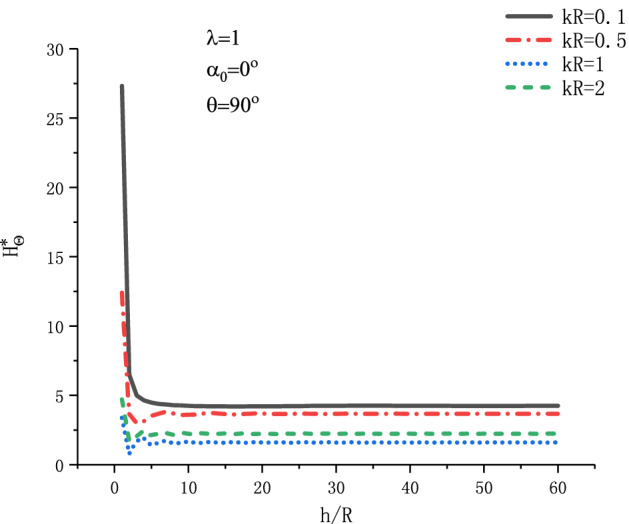
Variation of $H_{\theta }^{{\text{*}}}$ vs. ${h \mathord{\left/ {\vphantom {h R}} \right. \kern-\nulldelimiterspace} R}$ when SH wave incident horizontally.

Figure 21 shows the variation of $H_{\theta }^{{\text{*}}}$ at position $\theta  = {\pi  \mathord{\left/ {\vphantom {\pi  {\text{2}}}} \right. \kern-\nulldelimiterspace} {\text{2}}}$ with respect to ${h \mathord{\left/ {\vphantom {h R}} \right. \kern-\nulldelimiterspace} R}$ when SH wave is incident horizontally under different $kR$. Four values of incident frequency $kR$ have been discussed respectively in the Fig. 21. As can be seen from Fig. 21, with the increase of ${h \mathord{\left/ {\vphantom {h R}} \right. \kern-\nulldelimiterspace} R}$, the value of $H_{\theta }^{{\text{*}}}$ decreases abruptly and gradually becomes stable. This shows that when the circular cavity is farther and farther away from the horizontal boundary, the influence of the boundary on the MFICF at the edge of the cavity becomes smaller and smaller.

## Conclusions

In this paper, the problem of scattered stress and magnetic field intensity concentration of the SH wave caused by the circular cavity near the horizontal boundary of the rare earth giant magnetostrictive medium are studied by using the elastic wave theory and complex variable function. Through a specific example, the following conclusions can be obtained:

1. The factors such as incident wave frequency, incident angle, material geometrical parameter, permeability and the distance between the horizontal boundary and the circular cavity have influence on the DSCF and MFICF around the circular cavity.
2. When the SH wave is horizontally incident, in the case of low frequency, the DSCF and MFICF will get the maximum value. With the increase of frequency, the DSCF and MFICF tend to be stable.
3. When the wave incident vertically, the DSCF and MFICF around the circular cavity change more strongly as the case of the increase of frequency.
4. When the distance between the horizontal boundary and the circular cavity reaches a certain distance, the influence of the boundary on DSCF and the MFICF around the circular cavity will tend to be fixed.
5. The magnetic field intensity concentration factor increases at first and then decreases with respect to the piezomagnetic coefficient and permeability.

The present study is carried out under specific conditions, but the exact solution of the scattering of SH wave by circular cavity in semi-space rare earth giant magnetostrictive medium is presented, which can provide a reference for the approximate method of this problem. In addition, with reference to the excellent performance of the research method in this paper in the damage analysis of elastic materials and piezoelectric materials, the damage analysis of rare earth giant magnetostrictive materials in the future can be done by adding the repeated image method to deal with the strip plate model, or by adding the Green’s function method to study the two-phase magnetostrictive material model.
